# Assessment of Lean body mass using whole-body low-dose Ct from Pet/Ct to identify cancer Cachexia

**DOI:** 10.1186/s40644-026-01047-3

**Published:** 2026-05-23

**Authors:** G. J. O’Keefe, C. Senko, S. T. Lee, S. J. Gong, S. Pillai, R. Fogliaro, T. Chen, G. Ninatti, K. Pathmaraj, Z. Cao, I. Burvenich, L. Osellame, N. Hoogenraad, A. M. Scott

**Affiliations:** 1 Department of Molecular Imaging and Therapy, Austin Hospital, 145 Studley Road, Heidelberg, Melbourne, VIC 3084 Australia; 2 Olivia Newton-John Cancer Research Institute, Heidelberg, VIC 3084 Australia; 3 School of Cancer Medicine, Latrobe University, Bundoora, VIC 3086 Australia; 4 Department of Medicine, University of Melbourne, Parkville, VIC 3052 Australia

**Keywords:** Cachexia, Lean Body Mass (LBM), DEXA, PET/CT

## Abstract

**Introduction:**

Cancer cachexia is a multifactorial syndrome characterised by involuntary weight loss and muscle wasting, commonly seen in patients with advanced malignancies. Lean Body Mass (LBM) assessment is crucial for the early indentification and monitoring of cancer cachexia. Dual-energy X-ray absorptiometry (DEXA) is widely accepted as the gold standard for evaluating body composition. This study aims to develop a method for estimating LBM using whole-body low-dose CT acquired during PET/CT imaging.

**Methods:**

A cohort of patients enrolled in a prospective pilot study on cancer cachexia were included in the analysis. All patients underwent both DEXA and [^18^F]FDG PET/CT imaging. Lean body mass was assessed by calculating the Total Body Lean Mass ratio (TBLMr) from DEXA (TBLMr_DEXA_) and also whole-body low-dose CT (TBLMr_CT_).

**Results:**

A total of 32 (13-cachectic, 19-non-cachectic) patients with advanced malignancies were included in the analysis. The TBLMr_CT_ and TBLMr_DEXA_ were highly correlated (R^2^ = 0.92). Bland-Altman plots indicated a scaling bias with increasing TBLMr_DEXA_ values, with the non-cachectic group exhibiting a tighter distribution (0.06 ± 0.05) compared to the cachectic group (0.03 ± 0.11). TBLMr values were significantly different between cachectic and non-cachectic groups for both the DEXA-derived (*p* < 0.004) and low-dose CT-derived (*p* < 0.010) measurements.

**Conclusion:**

Whole-body low-dose CT obtained from routine [^18^F]FDG PET/CT imaging can provide an accurate estimate of LBM, showing high concordance with DEXA-derived measurements and clinical diagnosis of cachexia. This method offers a practical method for monitoring cancer cachexia in patients undergoing routine [^18^F]FDG PET/CT scans.

## Introduction

Cancer cachexia is a multifactorial syndrome characterised by the progressive, involuntary loss of body weight and skeletal muscle mass, often accompanied by adipose tissue loss [1, 2]. Cachexia is highly prevalent among patients with advanced-stage malignancies and is associated with increased morbidity, mortality, and a significant decline in quality of life [3]. Despite its clinical impact, cancer cachexia is frequently underdiagnosed and difficult to monitor, largely due to the lack of standardised diagnostic criteria and the limited availability of practical tools to assess body composition in routine oncology care [4].

One of the hallmarks of cancer cachexia is the loss of LBM, particularly skeletal muscle [5]. Early identification and monitoring of LBM changes are essential for timely diagnosis, clinical management, and tracking cachexia progression [1]. However, LBM is not routinely measured in cancer patients, partly because standard assessment methods are often time-consuming, impractical, or not readily available.

Dual-energy X-ray absorptiometry (DEXA) is widely recognised as the gold standard for body composition analysis, providing accurate quantification of fat, lean tissue, and bone mass [6, 7]. Nonetheless, DEXA is most commonly used in research settings and is not often included in standard clinical evaluations for oncology patients, due to the need of performing a separate dedicated scan.

Diagnostic computed tomography (CT), which is routinely performed for staging and follow-up in cancer patients, has been proposed as a more accessible and convenient alternative to DEXA for estimating LBM [8, 9]. Previous studies have shown that the L3 Skeletal Muscle Index, a surrogate measure of LBM derived from a single axial CT slice at the L3 vertebra level, might be a useful marker of sarcopenia and cancer cachexia [10–12]. However, single-slice or regional methods may not fully capture changes in whole-body muscle mass.

With the increasing use of PET/CT in oncology, there is now high availability of whole-body low-dose CT images, which are routinely acquired together with PET images for attenuation correction and anatomical localisation. This offers the opportunity to use whole-body low-dose CT to extract body composition data without requiring additional examinations. Recent studies have demonstrated the feasibility of using low-dose CT for estimating body fat and lean tissue distribution [13–15], providing a practical and potentially more comprehensive alternative to the L3 Skeletal Muscle Index.

This study aimed to develop and validate a reproducible approach for estimating LBM from routine low-dose CT acquired as part of PET/CT scans, using DEXA and clinical assessment as the reference standard, and to explore its potential as a practical, non-invasive tool for identifying cancer cachexia in patients with advanced malignancies.

## Materials and methods

### Study design and patient population

This study was conducted using data from a prospective pilot bioimaging trial conducted at Austin Health (Melbourne, VIC, Australia), which investigated [¹⁸F]FDG PET and DEXA as imaging biomarkers of cancer cachexia (NCT04127981 – Registration date 28th March 2019). Participants included patients with advanced non-small cell lung cancer, colorectal cancer, or pancreatic cancer undergoing standard of care [¹⁸F]FDG PET/CT imaging. Each patient was classified as cachectic or non-cachectic according to standard diagnostic criteria and underwent DEXA scanning within seven days of the [^18^F]FDG PET/CT, as part of the study protocol. Eligible patients were ≥ 18 years old, had an ECOG performance status of 0–2, and a life expectancy of at least four months. Cachexia was defined as unintentional weight loss > 5% over six months, or > 2% in patients with a BMI < 20 kg/m^2^ or with documented sarcopenia [2]. Exclusion criteria included uncontrolled diabetes mellitus, pregnancy, current use of medications interacting with the sympathetic nervous system, and conditions impairing the patient’s ability to undergo imaging procedures. All participants provided written informed consent for participation in the study and prior to each imaging assessment. Ethics approval for the study was obtained from the Austin Health Human Research Ethics Committee, Austin Radiation Sub-committee, and the Victorian Department of Human Services Radiation Safety Unit, and was conducted in accordance with the Declaration of Helsinki.

### DEXA acquisition and analysis

Each participant underwent a single DEXA scan using a Hologic Horizon scanner (Hologic Inc., Bedford, MA, USA), operating at endpoint energies of 100 & 140 kVp with a tube current of 2.5 mA.

DEXA analysis utilizes a three-compartment tissue model consisting of bone, lean tissue, and fat tissue. The measured x-ray attenuation coefficient µ_Total_(xy, E) at planar position xy and end-point energy E can be expressed in terms of the photo-absorption cross section for the Bone and Tissue class and their respective volumes class as follows:

$$\begin{aligned} {\mu _{Total}}\left( {xy,{\text{ }}E} \right){\text{ }} &= {\mu _{Bone}}\left( {xy,{\text{ }}E} \right){\text{ }} + {\mu _{Tissue}}\left( {xy,{\text{ }}E} \right){\text{ }} \\ &= {\sigma _{Bone}}\left( {xy,{\text{ }}E} \right){\rho _{Bone}}\left( {xy} \right){\text{ }} + {\sigma _{Tissue}}\left( {xy,{\text{ }}E} \right){\rho _{Tissue}}\left( {xy} \right) \hfill \\ \end{aligned} $$

In regions with no Bone content, the Tissue component can be further delineated into Lean and Fat Tissue classes:

$$\begin{aligned} {\mu _{Tissue}}\left( {xy,{\text{ }}E} \right){\text{ }} &= {\sigma _{Tissue}}\left( {xy,{\text{ }}E} \right){\rho _{Tissue}}\left( {xy} \right){\text{ }} \\ &= {\sigma _{Lean}}\left( {xy,{\text{ }}E} \right){\rho _{Lean}}\left( {xy} \right){\text{ }} + {\sigma _{Fat}}\left( {xy,{\text{ }}E} \right){\rho _{Fat}}\left( {xy} \right) \hfill \\ \end{aligned} $$

By measuring at two separate energies with the lower energy being below the K_α,β_ absorption energy for bone, this results in operational equations that are used to delineate the individual compartments into Bone and Tissue:

$$\rm \begin{aligned} & {\mu _{Total}}\left( {xy,{\text{ }}{E_1}} \right){\text{ }} = {\sigma _{Bone}}\left( {xy,{\text{ }}{E_1}} \right){\rho _{Bone}}\left( {xy} \right){\text{ }} + {\sigma _{Tissue}}\left( {xy,{\text{ }}{E_1}} \right){\rho _{Tissue}}\left( {xy} \right) \\ \end{aligned} $$

$$\rm \begin{aligned} & {\mu _{Total}}\left( {xy,{\text{ }}{E_2}} \right){\text{ }} = {\sigma _{Bone}}\left( {xy,{\text{ }}{E_2}} \right){\rho _{Bone}}\left( {xy} \right){\text{ }} + {\sigma _{Tissue}}\left( {xy,{\text{ }}{E_2}} \right){\rho _{Tissue}}\left( {xy} \right) \\ \end{aligned} $$

and similarly for Lean and Fat tissue for regions without bone:

$$\rm \begin{aligned} & {\mu _{Total}}\left( {xy,{\text{ }}{E_1}} \right){\text{ }} = {\sigma _{Lean}}\left( {xy,{\text{ }}{E_1}} \right){\rho _{Lean}}\left( {xy} \right){\text{ }} + {\sigma _{Fat}}\left( {xy,{\text{ }}{E_1}} \right){\rho _{Fat}}\left( {xy} \right) \\ \end{aligned} $$

$$\begin{aligned} & {\mu _{Total}}\left( {xy,{\text{ }}{E_2}} \right){\text{ }} = {\sigma _{Lean}}\left( {xy,{\text{ }}{E_2}} \right){\rho _{Lean}}\left( {xy} \right){\text{ }} + {\sigma _{Fat}}\left( {xy,{\text{ }}{E_2}} \right){\rho _{Fat}}\left( {xy} \right) \\ \end{aligned} $$

For the bone regions, the tissue components are delineated into Lean and Fat values based on neighbouring pixel values where bone is not present.

From the resultant report generated by the Hologic system, the Total Body Fat ratio (TBFr_DEXA_) and Total Body Lean Mass ratio (TBLMr_DEXA_) were derived from the %Fat and %Lean values and used for subsequent analysis.

**Fig. 1:**
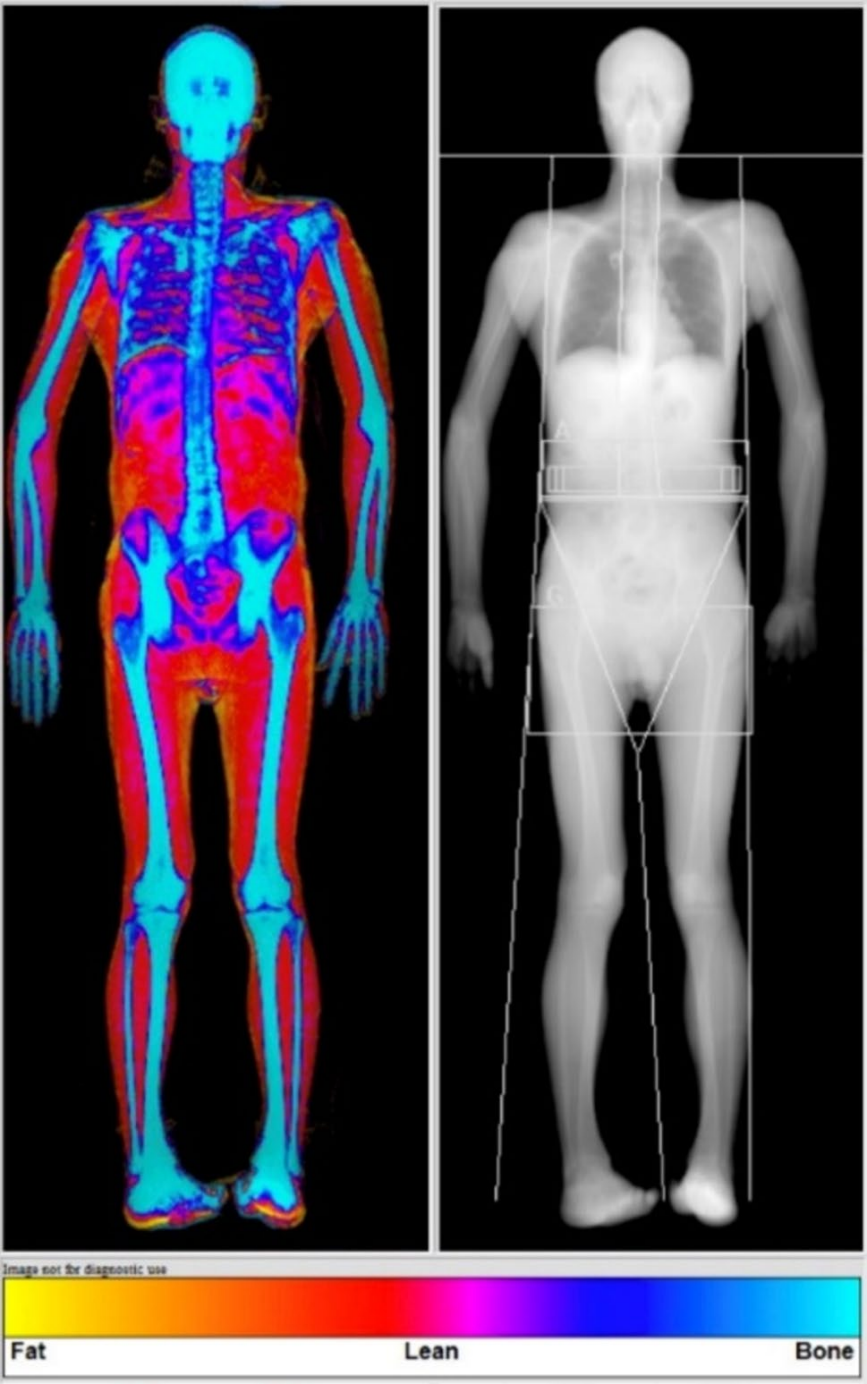
DEXA-determined tissue classification for subject AUS-001

An example of DEXA analysis and report are shown in Fig. 1; Table 1.

**Table 1:** An example of Hologic DEXA-generated report

Region	Area [cm2]	BMC [g]	BMD [g/cm2]	Fat [g]	Lean [g]	Lean + BMC [g]	Total [g]	%Fat [%]	%Lean [%]
L Arm	263.88	220.65	0.836	904	2768.7	2989.4	3893.4	23.2	71.1
R Arm	274.28	244.30	0.891	1072	3222.7	3467.0	4539.0	23.6	71.0
L Ribs	155.53	97.89	0.629						
R Ribs	113.15	100.38	0.887						
T Spine	169.93	141.76	0.834						
L Spine	70.77	76.10	1.075						
Pelvis	229.90	279.66	1.216						
Trunk		695.79		7049	26921.2	27617.0	34666.0	20.3	77.7
L Leg	390.63	598.82	1.533	2424	7530.6	8129.4	10553.4	23.0	71.4
R Leg	406.22	559.13	1.376	2339	7618.7	8177.8	10516.8	22.2	72.4
Sub Total	2074.29	2318.68	1.118	13,788	48062.0	50380.7	64168.7	21.5	74.9
Head	238.69	554.54	2.323	1278	2843.9	3398.4	4676.4	27.3	60.8
**Total**	**2312.98**	**2873.22**	1.242	**15,065**	**50905.9**	**53779.1**	**68844.1**	**21.9**	**73.9**

### PET/CT acquisition and whole-body low-dose CT analysis

All patients underwent a standard of care [^18^F]FDG PET/CT scan. Imaging was performed 60–70 min after injection of 220–350 MBq of [^18^F]FDG on a Phillips Ingenuity™ PET/CT camera (Philips Medical System, Cleveland, OH). The low-dose CT was acquired first, followed by the PET scan.

The low-dose CT component of the PET/CT extended from the skull vertex to mid-thigh, covering approximately 60% of the patient’s height. Low-dose CT images were acquired in spiral mode, with a rotation time of 0.5 s, a modulated tube-current of 80 mA, and a tube-voltage of 120 kVp, resulting in a dose-length product (DLP) distribution of 261 ± 44 mGy·cm.

A Hounsfield Unit (HU) window from − 250 HU to -50 HU was defined as the Fat Window and applied to the low-dose CT images based on the work by Borkan et al. [16], so that only voxels within this window range were used in tissue volume calculations to give an estimate of the Total Body Fat Volume (TBFV_CT_). By applying a tissue threshold of -300 HU and using voxels exceeding that value, a measure of the Total Body Volume (TBV_CT_) was determined.

The Total Body Fat Ratio (TBFr_CT_) was then calculated using the expression:

$$\rm TBF{r_{CT}} = {\text{ }}TBF{V_{CT}}/{\text{ }}TB{V_{CT}}$$

Similarly, Total Body Bone Volume (TBBV_CT_) was determined using a window from 250 HU to 3000 HU, and the Total Body Bone ratio (TBBr_CT_) was calculated.

The Total Body Lean Mass ratio (TBLMr_CT_) was derived as the complement of the fat and bone components, using the formula:

$$\rm TBLM{r_{CT}} = 1 -TBF{r_{CT}} -TBB{r_{CT}}$$

**Fig. 2:**
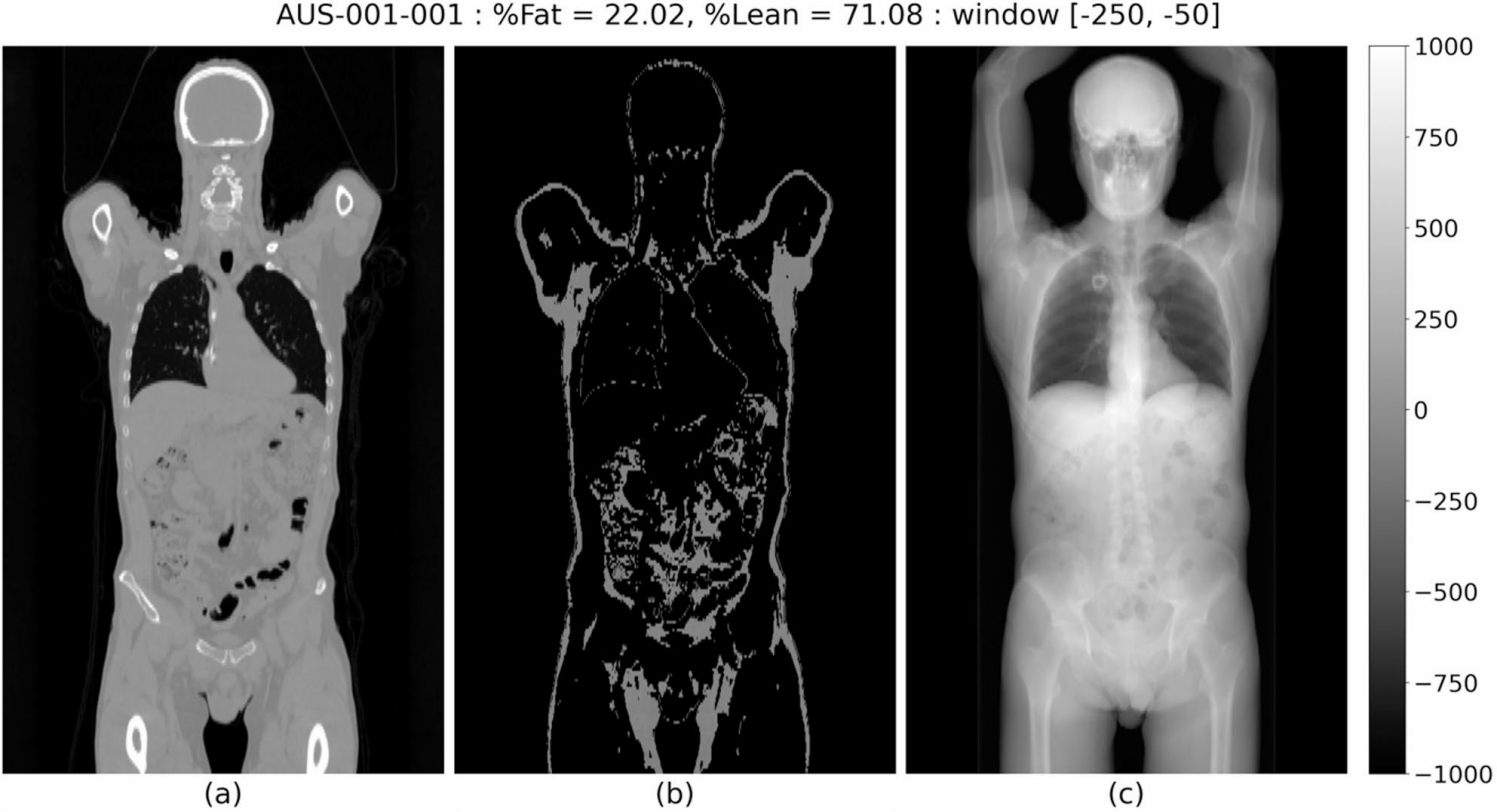
Low-dose CT of subject AUS-001, with **a**) whole-body tissue threshold (-300 HU); **b**) fat window (from − 250 HU to -50 HU); **c**) maximum intensity projection (MIP)

An example of low-dose CT analysis is shown in Fig. 2.

### Statistical analysis

Excel ^®^ (Microsoft^®^, Redmond, WA) was used to perform the statistical analysis. Patient demographic and clinical characteristics were summarized using standard descriptive statistics. Continuous variables were reported as means with standard deviations, while categorical variables were presented as frequencies. Correlation between DEXA- and low-dose CT-derived TBLMr values was evaluated using linear regression. Differences in TBLMr values between cachectic and non-cachectic groups were assessed using one-tailed Student’s t-test. Bland-Altman plots were generated to evaluate the degree of agreement, bias, and variability across measurements. Proportional bias was assessed by examining whether discrepancies between DEXA- and low-dose CT-derived TBLMr values increased with higher patient’s TBLMr values. Diagnostic accuracy of DEXA- and low-dose CT-derived TBLMr values for detecting cachexia was assessed using receiver operating characteristic (ROC) curve analysis and area under the curve (AUC) calculation. P values < 0.05 were considered statistically significant.

## Results

A total of 33 patients were enrolled in the cancer cachexia pilot bioimaging trial between 2019 and 2023. The cohort included 16 males and 17 females, with a mean age of 60 years. One participant did not complete the study protocol, leaving 32 subjects eligible for the analysis. Patients were evenly distributed across tumour types, with 11 diagnosed with advanced non-small cell lung cancer, 10 with advanced colorectal cancer, and 11 with advanced pancreatic cancer. At the time of analysis, 13 patients met the diagnostic criteria for cancer cachexia, while the remaining 19 were classified as non-cachectic. Table 2 provides an overview of patient demographics and clinical characteristics.

**Table 2:** Demographic and clinical characteristics of patients included in the analysis

	sex	height cm	weight kg	age yr	clinical diagnoses	
AUS-001	M	184	68.7	56	cachectic	pancreatic
AUS-002	M	175	50	54	cachectic	pancreatic
AUS-003	F	168	52.56	73	cachectic	pancreatic
AUS-004	M	176	55	76	cachectic	pancreatic
AUS-005	M	178	74	62	cachectic	pancreatic
AUS-006	M	180	76	69	cachectic	pancreatic
AUS-007	F	160	64	61	non-cachectic	pancreatic
AUS-008	F	159	68.8	57	non-cachectic	colorectal
AUS-009	F	152	61	45	non-cachectic	colorectal
AUS-010	F	169	72	45	non-cachectic	colorectal
AUS-011	F	163	73	70	non-cachectic	colorectal
AUS-012	F	164	64	53	non-cachectic	colorectal
AUS-013	F	174	68	51	non-cachectic	colorectal
AUS-014	F	152	60	37	non-cachectic	colorectal
AUS-015	F	159	63	59	non-cachectic	colorectal
AUS-016	F	161	70	62	non-cachectic	pancreatic
AUS-017	F	165	77	53	cachectic	pancreatic
AUS-018	M	169	79	70	non-cachectic	lung
AUS-019	M	169	79	64	non-cachectic	lung
AUS-020	M	168	78	63	non-cachectic	lung
AUS-021	F	158	52	47	non-cachectic	lung
AUS-022	F	164	62	76	non-cachectic	lung
AUS-023	M	174	65	68	non-cachectic	lung
AUS-024	M	164	68	56	non-cachectic	lung
AUS-025	F	147	65.5	64	cachectic	lung
AUS-026	M	173	77	63	cachectic	pancreatic
AUS-027	M	173	66.5	62	non-cachectic	lung
AUS-028	M	180	67	54	cachectic	lung
AUS-029	M	174	84	65	cachectic	colorectal
AUS-030	F	162	65	54	cachectic	pancreatic
AUS-031	M	170	49.2	69	cachectic	colorectal
AUS-032	M	173	89	54	cachectic	colorectal
AUS-033	F	157	71	63	non-cachectic	lung
average						
all	33	167	68	60		
male	16	174	70	61		
female	17	161	65	58		
stdev						
all		8.82	9.56	9.73		

TBLMr values derived from DEXA and low-dose CT are shown in Table 3.

**Table 3:** TBLMr values obtained from DEXA and low-dose CT

Non-cachexia group Total Body Lean Mass Ratio				Cachexia group Total Body Lean Ratio		
	DEXA	ldCT			DEXA	ldCT
**AUS-007**	0.562	0.489		**AUS-001**	0.739	0.711
**AUS-008**	0.536	0.458		**AUS-002**	0.771	0.832
**AUS-009**	0.556	0.480		**AUS-003**	0.571	0.606
**AUS-010**	0.541	0.447		**AUS-004**	0.775	0.824
**AUS-011**	0.554	0.483		**AUS-005**	0.684	0.654
**AUS-012**	0.558	0.506		**AUS-006**	0.642	0.578
**AUS-013**	0.571	0.489		**AUS-017**	0.587	0.467
**AUS-014**	0.562	0.455		**AUS-025**	0.486	0.396
**AUS-015**	0.547	0.451		**AUS-026**	0.678	0.652
**AUS-016**	0.557	0.521		**AUS-028**	0.715	0.725
**AUS-018**	0.518	0.434		**AUS-029**	0.666	0.629
**AUS-019**	0.601	0.575		**AUS-030**	0.526	0.484
**AUS-020**	0.650	0.613		**AUS-031**	0.757	Not Acquired
**AUS-021**	0.664	0.649		**AUS-032**	0.618	0.515
**AUS-022**	0.557	0.490				
**AUS-023**	0.707	0.691				
**AUS-024**	0.625	0.547				
**AUS-027**	0.654	0.610				
**AUS-033**	0.545	0.502				
**mean**	0.582	0.521			0.651	0.621
**stdev**	0.052	0.072			0.091	0.133
**N**	19	19			14	13
**statistical analysis : Non-cachexia vs. cachexia groups**						
**statistic**	**DEXA**	**ldCT**				
t-stat	2.786	2.470				
p-value	0.004	0.010				

**Fig. 3:**
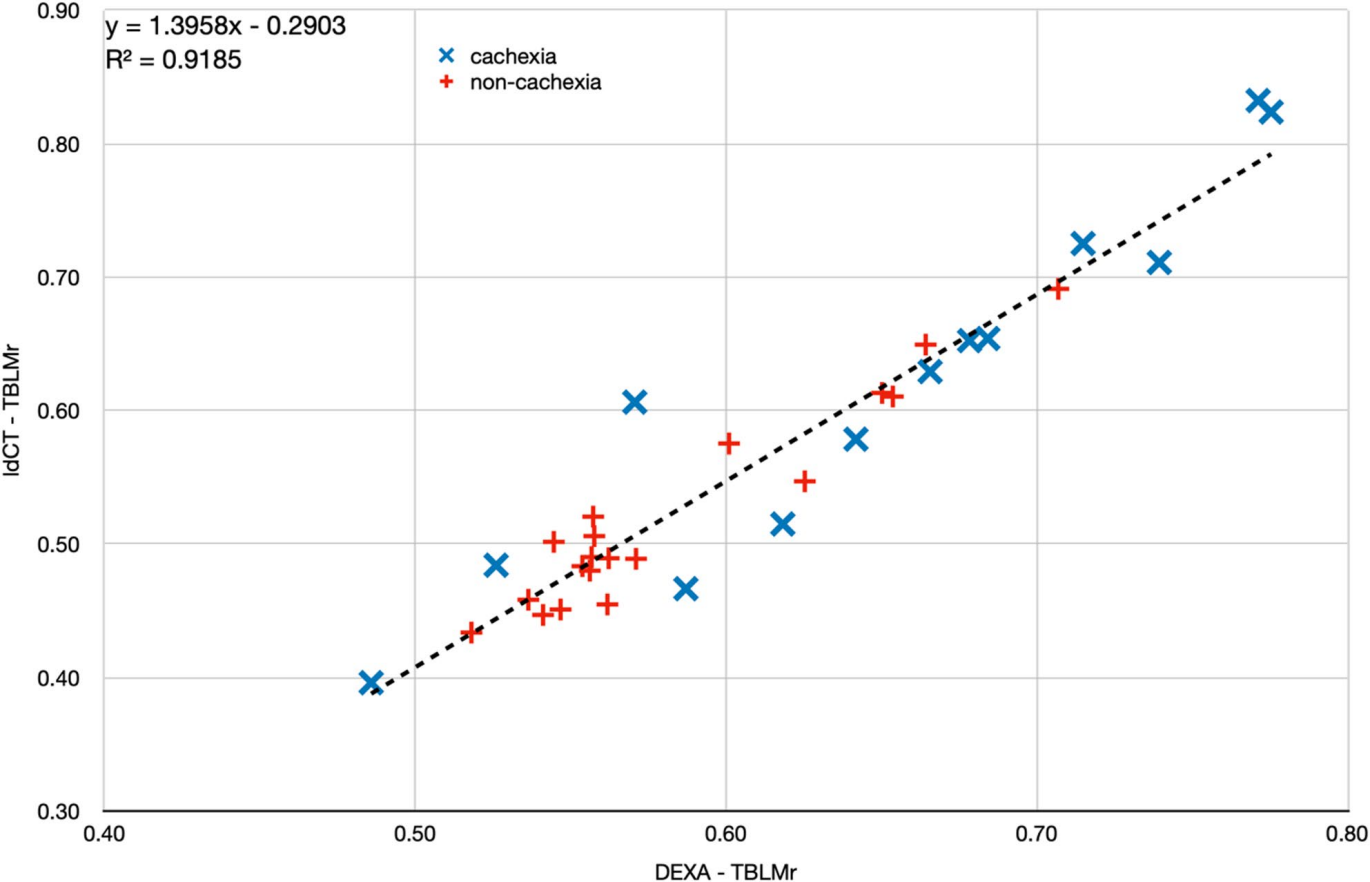
Linear regression analysis comparing TBLMr_CT_ and TBLMr_DEXA_

The linear regression analysis comparing TBLMr_CT_ and TBLMr_DEXA_ is shown in Fig. 3. The regression revealed that the TBLMr values derived from low-dose CT were, on average, approximately 40% higher than those obtained from DEXA.

TBLMr values were significantly different between cachectic and non-cachectic patients for both DEXA (*p* = 0.004) and low-dose CT-derived (*p* = 0.010) measurements.

**Fig. 4:**
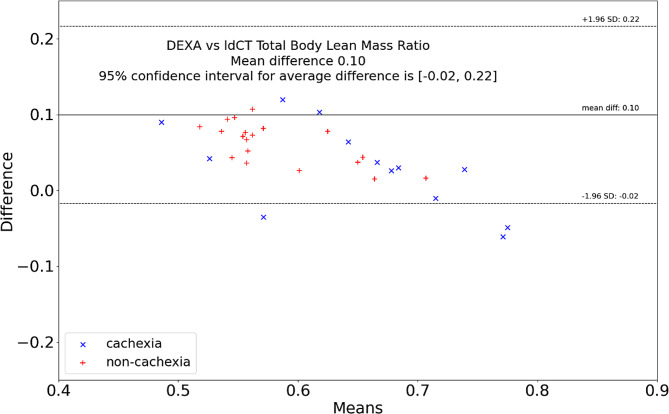
Bland-Altman plot comparing TBLMr_CT_ and TBLMr_DEXA_ in cachectic and non-cachectic patients

Bland-Altman plots for the cachectic, non-cachectic, and combined groups are presented in Fig. 4. Across all groups, differences between DEXA- and low-dose CT-derived TBLMr values fell within the two standard deviation range. A small mean bias was observed, 0.03 for the cachectic group and 0.06 for the non-cachectic group. A proportional bias was noted in both groups, indicating that for increasing TBLMr values the discrepancy between the DEXA- and low-dose CT-derived TBLMr was larger. The non-cachectic group showed tighter clustering, with a mean difference range between DEXA and CT-derived TBLMr of 0.06 ± 0.05, compared to 0.03 ± 0.11 in the cachectic group.

**Fig. 5:**
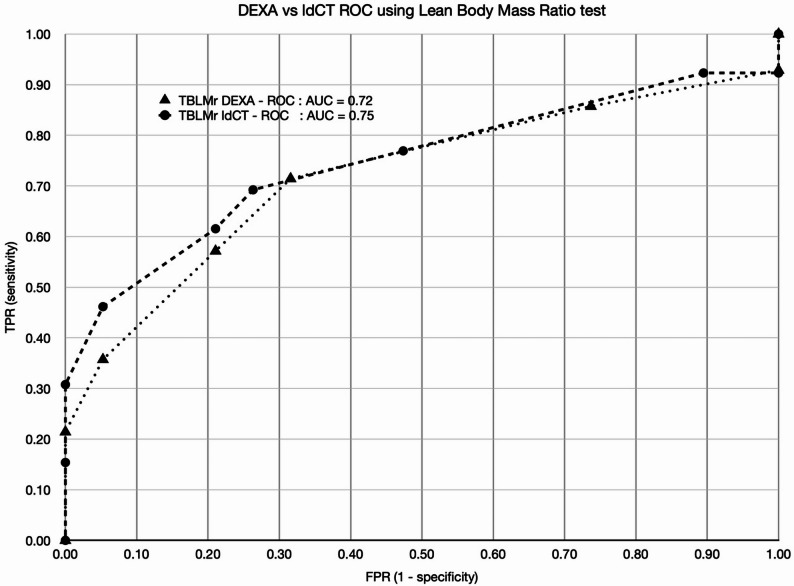
ROC curves for TBLMr_CT_ and TBLMr_DEXA_

Results of the ROC curves analysis for DEXA and low-dose CT are shown in Fig. 5. The ROC AUC was 0.72 for TBLMr_DEXA_ and 0.75 for TBLMr_CT_, suggesting that the two methods have comparable sensitivity and specificity for identifying cancer cachexia.

## Discussion

This study demonstrates that whole-body low-dose CT, routinely acquired during [¹⁸F]FDG PET/CT imaging, is able to estimate body composition with comparable accuracy to DEXA. Low-dose CT may therefore be used as a non-invasive and practical tool for assessing LBM and supporting the diagnosis and montitoring of cancer cachexia.

Our study showed that patients with cancer cachexia had significantly higher TBLMr values compared to non-cachectic patients, both on low-dose CT and DEXA-derived measurements. Our findings support the feasibility of using whole-body low-dose CT from PET/CT scans performed in oncology patients as a practical alternative to DEXA, with the advantage that no additional dedicated imaging is required. The method we have used may also be suitable for automated / AI analysis approaches, thus making this approach more generally applicable to routine PET/CT studies.

To our knowledge, this is the first study to investigate the potential of using low-dose CT from PET/CT imaging for body composition assessment in a well-controlled cohort of cancer patients with and without cachexia, using DEXA as the gold standard comparator.

Previous studies have explored the use of low-dose CT from PET/CT to assess body composition in other settings, Sarikawa et al. [17, 18] used LBM estimates to provide a LBM corrected Standard Uptake Value (SUV), which they refer to as SUL, for 18 F-FDG scans to address the over-estimation SUV in obese patients. Miller et al. [14] used a combination of deep learning and image processing to automatically quantify skeletal muscle, bone, and adipose tissue in patients undergoing PET/CT myocardial perfusion imaging. They found that body composition data could be rapidly extracted from low-dose CT and that increased adipose tissue density, particularly in visceral fat, was associated with a higher risk of death or myocardial infarction. Similarly, Lee et al. [15] evaluated body composition using whole-body low-dose CT from PET/CT in healthy adults, comparing their findings with bioelectrical impedance analysis and muscle strength tests. They observed a strong correlation between low-dose CT-derived body composition parameters and bioelectrical impedance analysis and muscle strength tests. Moreover, they found that whole-body CT-extracted parameters had a more significant association with bioelectrical impedance analysis and muscle strength tests than regional assessments limited to the abdominal region, suggesting that whole-body analysis may offer a more accurate and comprehensive evaluation of sarcopenia and cachexia.

Our study has some limitations. Firstly, the cohort size was relatively small, and there was a slight imbalance between cachectic (*n* = 13) and non-cachectic (*n* = 19) groups. Additionally, body composition analysis was based on low-dose CT images covering the area from the skull vertex to mid-thigh, which is the standard field of view for PET/CT imaging. This might have affected the accuracy of the measurements, as lower limbs were not included. Expanding PET/CT imaging to include the entire body would require longer scan times, which is often not feasible in many centres. Moreover, the likelyhood that a full body PET/CT scan would improve accuracy of body composition parameters is not certain. However, with the growing availability of large field-of-view PET/CT scanners able to cover the entire body rapidly, full-body imaging may become feasible in the near future without significantly increasing acquisition times [19, 20].

## Conclusion

This study demonstrates that whole-body low-dose CT acquired as part of PET/CT imaging can be used to assess body composition and estimate LBM, providing a practical alternative for the detection and monitoring of cachexia in patients with advanced malignancies undergoing longitudinal PET/CT measurements.

## Data Availability

Available on request.
